# Polysaccharide metabolism regulates structural colour in bacterial colonies

**DOI:** 10.1098/rsif.2022.0181

**Published:** 2022-05-25

**Authors:** Gea T. van de Kerkhof, Lukas Schertel, Laura Catòn, Thomas G. Parton, Karin H. Müller, Heather F. Greer, Colin J. Ingham, Silvia Vignolini

**Affiliations:** ^1^ Department of Chemistry, University of Cambridge, Lensfield Road, Cambridge CB2 1EW, UK; ^2^ Hoekmine BV, Room 1.091 (iLab), Kenniscentrum Technologie en Innovatie, Hogeschool Utrecht, Heidelberglaan 7, 3584 CS, Utrecht, The Netherlands; ^3^ Cambridge Advanced Imaging Centre, University of Cambridge, Downing Street, Cambridge CB2 3DY, UK

**Keywords:** metabolism, structurally coloured bacteria, fucoidan, polysaccharides, biochemical pathway, regulation

## Abstract

The brightest colours in nature often originate from the interaction of light with materials structured at the nanoscale. Different organisms produce such coloration with a wide variety of materials and architectures. In the case of bacterial colonies, structural colours stem for the periodic organization of the cells within the colony, and while considerable efforts have been spent on elucidating the mechanisms responsible for such coloration, the biochemical processes determining the development of this effect have not been explored. Here, we study the influence of nutrients on the organization of cells from the structurally coloured bacteria *Flavobacterium* strain IR1. By analysing the optical properties of the colonies grown with and without specific polysaccharides, we found that the highly ordered organization of the cells can be altered by the presence of fucoidans. Additionally, by comparing the organization of the wild-type strain with mutants grown in different nutrient conditions, we deduced that this regulation of cell ordering is linked to a specific region of the IR1 chromosome. This region encodes a mechanism for the uptake and metabolism of polysaccharides, including a polysaccharide utilization locus (PUL operon) that appears specific to fucoidan, providing new insight into the biochemical pathways regulating structural colour in bacteria.

## 1. Introduction

Structural colour can be produced by nanostructures with dimensions on the same scale as the wavelength of visible light. Because of the similarity in scale light reflected at the interface of such structures can constructively interfere, creating vibrant colours. These structural colours, in contrast to pigmented colours generated by selective absorption of light, appear in a wide variety of organisms from plants [1,2], to animals [3–5] and also in bacterial colonies [6–11].

While considerable effort has been spent on understanding the physical mechanisms responsible for structural coloration [12,13], less attention has been paid to the biochemical pathways involved in the development of such structures [14–16]. In this context, the regulatory pathways linking environmental conditions to the development of photonic structures should provide important insights into the biological function of structural colour in nature [17,18]. For example, environmental factors such as temperature and nutrient availability could be used in honesty signalling towards other organisms [17,19–23]. Therefore, a better understanding of such regulatory mechanisms could help to elucidate the development and function of structural colour in nature, and in turn facilitates the creation of new photonic structures, for example by genetic modification.

In bacterial colonies, where structural colour originates from the organization of the cells within the colony, the mechanisms that regulate development of structural colour remain unclear [24]. While it is known that individual cell properties such as gliding motility and the aspect ratio of the cells affect the coloration [25], no regulatory mechanisms have been elucidated, and research on the influence of external factors on the organization of the cells is limited [26,27]. A key parameter that has been largely overlooked is the influence of nutrient content. Nutrient conditions are of particular interest when studying structurally coloured bacterial strains, as they can directly link to the biological function of such photonic structures [28].

Here, we studied the effect of nutrients on the development of photonic structures in the structurally coloured bacteria strain *Flavobacterium* Iridescent 1 (IR1). In particular, we investigated the organization of IR1 in the presence and absence of fucoidan, a polysaccharide originating from brown algae, and compared the results to colonies grown on *κ*-carrageenan, a polysaccharide produced by red algae. We target such algal polysaccharides because it has been suggested that macroalgae might be a suitable surface supporting the organization of structurally coloured colonies [8,10,26,28]. Therefore, we speculate that algal products are likely to be a natural food source and habitat for these bacteria, and the IR1 strain has been shown to respond differently to the presence of red and brown algae [25]. A detailed optical characterization of the organization of the cells when grown under these different conditions is provided and correlated to a highly specific metabolic pathway, using mutant strains that no longer show the regulation of structural colour by growth on fucoidan.

Our results identify the presence of a metabolic pathway regulating structural colour in strain IR1, offering a unique perspective on the regulatory mechanisms involved in the development of structural colour in bacteria.

## 2. Results and discussion

### 2.1. Addition of fucoidan affects the optical properties of bacterial colonies

We first investigated the influence of two types of algal polysaccharides (fucoidan and *κ*-carrageenan) on the development of photonic structures in IR1 wild-type (WT) colonies. Fucoidan is a sulphated polysaccharide produced by brown algae, while *κ*-carrageenan is also a sulphated polysaccharide but originating from red algae. First, we compared the optical properties of colonies grown in the presence of fucoidan to those cultivated in the absence of any added polysaccharides (artificial seawater agar black (ASWB) with added fucoidan (ASWBF) and without added polysaccharides (ASWB), table 1), while in a second stage, we then compared these results to the development of IR1 in the presence of *κ*-carrageenan (ASWBC). In specific, we use 1% (w/v) fucoidan and 0.5% (w/v) *κ*-carrageenan in the agar plates, as those were determined to be the concentrations required for optimal coloration (electronic supplementary material, figure S1). For polysaccharide concentrations higher than 1%, coloration is inhibited, and for concentrations lower than 0.5% no effect of the polysaccharides can be observed. For fucoidan plates, a concentration of 1% was chosen rather than 0.5% because it gives a more stable purple coloration that lasts longer, facilitating the study of this optical effect (electronic supplementary material, figure S2).

**Table 1.** Composition of each type of agar plate used in this study.

name	components (per litre)
ASWB	17.5 g agar (Sigma, 9002-18-0), 10 g KCL (Fisher Chemical, 7447-40-7), 1 g yeast extract (Sigma, Y1625), 5 g peptone (Sigma, 70173), 0.33 g nigrosin (Sigma, N4763)
ASWBF	ASWB plate with the addition of 10 g fucoidan (Absonutrix)
ASWBC	ASWB plate with the addition of 5 g *κ*-carrageenan (Sigma, 22048)
ASW	ASWB plate without the addition of any nigrosin
ASWF	ASWBF plate without the addition of any nigrosin

**Figure 1.**
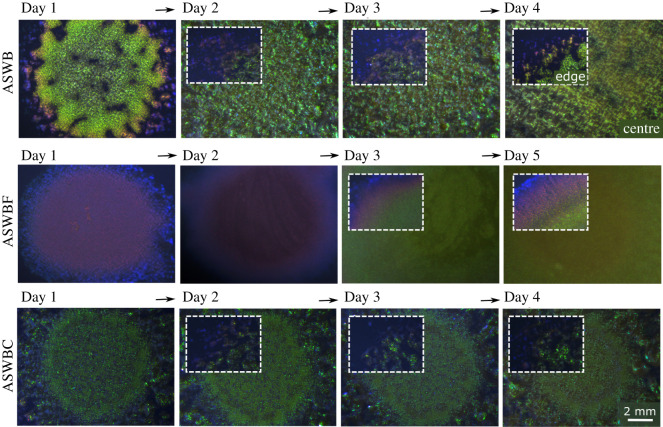
Colour development of IR1 colonies throughout their lifespan under different nutrient conditions: an ASWB plate without any added polysaccharides, an ASWBF plate with added fucoidan and an ASWBC plate with added *κ*-carrageenan. The images show the centre of each colony, at the location where the initial cell suspension was deposited. The insets show the edge of each colony, at the same scale as the main images.

As shown in figure 1, the nutrient conditions strongly influence both colour and the uniformity of colour within the colony in every stage of development. On ASWB agar plates, IR1 colonies exhibit sparkling green structural colour for several days after inoculation. Coloration first appears as a light green/yellow (peak wavelength 578 nm on day 1 after inoculation, electronic supplementary material, figure S3), then it shifts to a darker green (548 nm, day 2), before it gradually disappears from day 3 onwards. By contrast, on ASWBF plates, colonies show a very different colour development: coloration is dark purple on days 1 and 2 before becoming green on day 3. The shift from purple to green coloration is considered to stem from a local depletion of fucoidan, which is supported by a faster colour shift (from day 1 to day 2), when the concentration of fucoidan is lower (electronic supplementary material, figure S2). When viewing the cells in a disordered state, on an inoculation loop (electronic supplementary material, figure S4), it can be seen that the difference in coloration of IR1 grown on ASWB and ASWBF comes from changes in the organization of the cells, and not from a fucoidan-induced change in pigmentation of the cells or the extracellular matrix, as both samples have a similar appearance. The molecular properties of fucoidan that induce the change in the organization of the cells after the compounds are metabolized are still unknown. Investigating this would require a biochemical study that is out of the scope of this study. In contrast to the ASWBF plates, in the case of *κ*-carrageenan (ASWBC plates), we do not observe strong colour variations during development, with a consistent green appearance throughout the lifespan of the colony.

It is important to report that variations in colour are also usually observed within the same colony (see insets in figure 1), independently of the growth conditions. At the very edge of the colony, this corresponds to the spreading front, where the colony is relatively young. Cells are not as densely packed in this area as they are in older parts of the colony, resulting in a shift to a longer wavelength.

In addition to the colour change, the colour intensity and iridescent appearance of the colony also differ for the various nutrient conditions. While IR1 on ASWB plates has a brilliant iridescent appearance (figure 1; electronic supplementary material, figure S3) [11], the colony appears relatively dark and matte when grown in presence of fucoidan. By contrast, when grown on *κ*-carrageenan, the colony has a bright iridescent appearance, similar to the colonies grown in the absence of added polysaccharides. However, for ASWBC plates, there is a distinct difference in the angular dependency of the reflected light from the centre of the colony, which is defined as the place of initial inoculation, and the radially spread area. This difference is not present on ASWB plates, indicating a change in spreading behaviour between the two conditions.

To deepen our understanding on the correlation between the nutrient uptake, the nanoscale organization and the macroscopic appearance of the bacterial colonies, we then studied the optical response of the colony using an angular resolved spectroscopy set-up, which has previously been used to study the structural organization of bacterial colonies [10,11,25].

### 2.2. Optical characterization reveals changes in the structural organization

It is well known that the structural colour of IR1 colonies is the result of the organization of the bacterial cells into two-dimensional photonic crystals with hexagonal packing [11,25]. The peak wavelength of reflected light is determined by two factors: the refractive index contrast between the bacteria and surrounding medium, and the lattice constant *d* of the crystal structure, given by the inter-bacterial distance in the plane of hexagonal packing. Additionally, orientation and size of local crystalline domains, and any degree of disorder can strongly affect the colour homogeneity and intensity.

Therefore, to reveal the organization of the cells in the IR1 colonies, we use angular resolved spectroscopy in two measurement modes: (i) specular reflection mode, where the angle of observation always equals the angle of incidence and (ii) scattering mode, where the scattered light intensity over a range of angles is collected for a fixed illumination angle [29]. The two measurement modes provide complementary information about the optical properties of the structure: the specular signal provides the average refractive index of the sample (obtained using Snell’s Law corrected for the angle of incident light), while the diffraction peaks observed in the scattering signal provide the lattice constant *d* of the photonic structure [11]. Unless indicated otherwise, all the measurements were performed on the centre of each colony.

#### 2.2.1. Spectral shift of diffraction spots

Interestingly, from specular measurements of IR1 colonies we find that the average refractive index of the colony remains stable at a value of *n*_avg_ = 1.4 ± 0.05 across all nutrient conditions and all developmental stages (electronic supplementary material, figure S5). We thus conclude that the variation in colour over time and under different nutrient conditions does not stem from a change in the effective refractive index of the system, and must therefore be connected to changes in the structural organization of the colonies.

**Figure 2.**
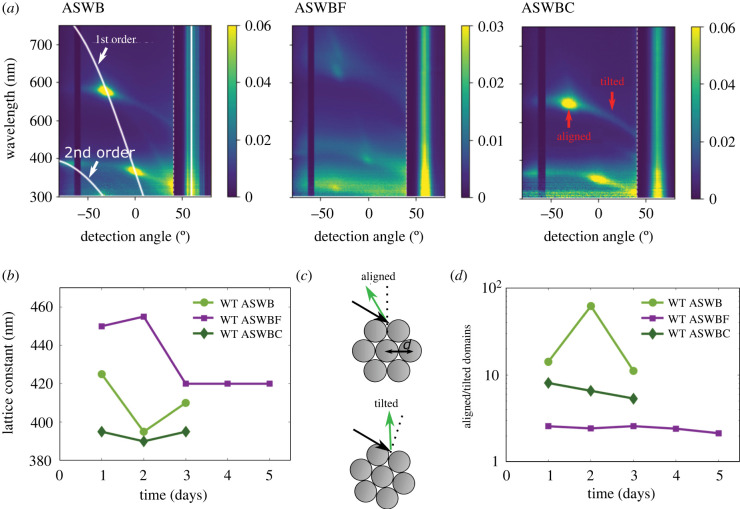
(*a*) Angular resolved spectral reflection in scattering mode for IR1 grown under three different nutrient conditions on day 1 of their growth. The illumination angle is kept at −60° for all measurements. Due to a limitation of the set-up, light cannot be detected at the angle of illumination, creating the dark band observed at −60°. Reflected light intensity is normalized against a white diffuser, and represented by a heat map, with blue low intensity and yellow high intensity. The angular range inside the white dotted lines contains the specular reflection angle (+60°), where the reflectance is much stronger than at other angles. We therefore show the reflectance in this angle range divided by a factor of 300 so that it can be displayed without saturating the colour scale. The solid white lines show a fit of the grating equation to the diffraction peaks, giving us a value for the lattice constant as shown in (*b*). (*b*) Development of the lattice constant for IR1 colonies grown under different nutrient conditions. (*c*) Schematic showing how the tilt angle of the crystalline domains determines the angle at which light is diffracted. (*d*) Development of the ratio between aligned and tilted domains over time, for different nutrient conditions.

Scattering measurements at a fixed illumination angle exhibit diffraction peaks, visible as high-intensity spots in the angular resolved spectral maps (see figure 2*a*; electronic supplementary material, figure S3). By fitting these diffraction peaks to the grating equation, we obtain precise values for the lattice constant, which provides information on the spacing between cells or their size (within an accuracy of 10 nm) [11]. Therefore, by performing scattering measurements on colonies grown under different nutrient conditions, we could track the evolution of the inter-bacterial distance over time (figure 2*b*).

As shown in figure 2*b*, colonies grown on ASWB plates have a lattice constant of *d* = 425 nm on day 1, corresponding to the light green colour observed in optical microscopy (figure 1), which decreases to *d* = 395 nm on day 2 when the colony has grown and the packing of the cells becomes denser. Finally, on day 3, the bacteria start losing their coloration, the offset of which is indicated by a loosening of their dense packing which leads to an increase of the lattice constant, to a value of *d* = 410 nm.

By contrast, for ASWBF plates, the lattice constant on day 1 is *d* = 450 nm, resulting in a red-shift of the reflected colour with respect to ASWB plates. It is important to highlight that the visual appearance of the fucoidan colony on day 1 (as captured by the photograph in figure 1), looks purple instead of red, because of the first-order diffraction spot at lower wavelengths. While the second order can not be perceived in the colony growing on an ASWB plate (as it is in the UV spectral region at 364 nm on ASWB day 1), on the ASWBF plate, it strongly contributes to the visual response (400 nm, ASWBF day 1, see electronic supplementary material, figure S6). Therefore, because of this combination of a red and blue diffraction spot, the colony has the observed purple appearance. The lattice constant increases slightly on day 2, to *d* = 460 nm, which could indicate further adaptation of the colony to fucoidan that occurs over time, or potentially an increased cell death. However, from the change to a stable green with *d* = 420 nm after day 2, which remains until a final loss of colour after day 5, it can be concluded that cell death is not widespread, because the bacteria are still actively changing their coloration to lower lattice constants. It is worth noting that the colony retains its colour much longer on fucoidan than under other conditions (the centre of the colony is coloured for 5 days on ASWBF versus 3 days on ASWB and ASWBC plates, figure 2*b*,*d*). The loss of colour indicates a decreased ordering off the cells, which could be caused by depletion of the nutrients and subsequent cell death. Moreover, colony expansion is much slower (it takes 7 days on ASWBF versus 3 days on ASWB and ASWBC plates for the colony to cover the entire surface of the agar, electronic supplementary material, figure S7a). This appears to be in accordance with the reported slow metabolism of fucoidan by microorganisms [30,31].

Finally, for ASWBC plates, the lattice constant was similar to the ASWB plates on day 2 (within the margin of error for this measurement), corresponding to a constantly close-packed colony. Contrary to the ASWB plates, the lattice constant remains unchanged across the colony’s lifespan.

Direct observation of the bacteria size on their second day of growth in cryogenic transmission electron microscopy (cryo-TEM) reveals that the increased lattice constant for colonies grown on fucoidan is a direct result of an increase in cell size (electronic supplementary material, figure S8). By studying cryo-TEM images of at least 40 cells grown on both ASWB and ASWBF plates, we measured an average cell diameter of 400 ± 20 nm for IR1 on ASWB plates, and 480 ± 25 nm for IR1 on ASWBF plates. This average diameter of 480 nm for cells on ASWBF plates is, surprisingly, larger than the lattice constant (460 nm). We believe that this is an artefact of the measurement, expressed by the asymmetric size distribution (electronic supplementary material, figure S8a) and potentially caused by swelling of the cells during sample preparation steps where the suspension is kept at low-salt concentration, which is done to avoid deposition of salt on the TEM grids that obscures the cells. However, regardless of slight deviations, it is clearly observed that fucoidan-grown cells have a significantly larger average diameter than cells grown on ASWB plates conditions.

In addition to a larger mean diameter, fucoidan-grown cells also exhibit greater variation in diameter along the length of each individual cell. The diameter of a cell is determined by taking the average of the values measured at set intervals of 300 nm across the length of the cell (see electronic supplementary material, figure S8b). However, by taking the average standard deviation of the values found within each cell, we find that fucoidan-grown cells have an average variation of 20 ± 10 nm across the length of their cells, whereas for cells on ASWB plates this is only 10 ± 4 nm.

Previous work has shown that cell length could potentially play a role in the optimization of the ordering of IR1 cells, with shorter cells resulting in better long-range correlation [25]. However, while cell diameter changes for different nutrient conditions, cell length remains approximately the same, with 3.2 ± 0.8 μm for fucoidan-grown cells and 3.4 ± 0.8 μm for cells grown on ASWB plates. Since IR1 cells multiply by lengthwise elongation and division, a large variation in cell length is expected and the difference between the two conditions found here is considered negligible.

It appears that the uptake and metabolism of fucoidan triggers a change in cell morphology, altering the photonic structure of the colony. The cell diameter must be regulated quite precisely to allow shifts in structural coloration but the mechanism is not known.

#### 2.2.2. Angular distribution of diffraction signals

Besides the shift in colour, the overall colour intensity and angular dependency of the reflected light are also affected by changes in the nutrient conditions (figure 2*a*).

The matte appearance of a colony (as seen for the ASWBF samples) can be attributed to a wider angular distribution in the orientation of crystalline domains within the colony. The tilt of the two-dimensional hexagonal packing of the bacteria determines the angle of reflection [10,11], as illustrated in figure 2*c*. If neighbouring crystalline domains are well aligned to the agar surface, they will all reflect light at the same angle, producing more intense iridescent colours at that angle. By contrast, if neighbouring domains have a range of tilts, they will reflect light at a range of different angles, resulting in a more matte appearance.

We can quantify the degree of domain alignment by considering the ratio of domains that are aligned to the surface of the agar (in-plane), to the ones that are tilted out of this plane. Lower values of this ratio correspond to a more matte appearance. As the majority of domains are aligned to the surface of the agar, the signal from these domains will contribute to what we define as the main diffraction peak. On the other hand, the tilted domains (i.e. not aligned to the agar surface) will contribute to the angular spread of the diffraction peak. As the measured intensity in a specific diffraction direction can be assumed to be proportional to the total volume of domains aligned in a specific direction, we use this measure to estimate the level of alignment. Specifically, we represent the domains aligned with the surface by the intensity value of the main diffraction peak at −30° ± 4°,^1^ and for the tilted domains the intensity value recorded at the angle of the main peak plus 55°. By dividing these two values, we therefore estimate the ratio between aligned and tilted domains: with a higher value indicating a more ordered colony. Repeating the same procedure for the different nutrient conditions, we note that colonies grown in the presence of fucoidan are much less ordered, with more tilted domains, compared to those grown on ASWB and ASWBC plates (figure 2*d*).

It should be noted, that only the measurements for ASWB days 1 and 2, and ASWBF day 2, have been repeated. For determining the lattice constant, derived from the wavelength and detection angle of the reflectance peak, this was sufficient as the error of this measurement was reproducible at ±5 nm. For determining the ratio between aligned and tilted domains, based on the intensity of the signal, the error was less consistent, with higher errors for the high-intensity spots. However, since ASWB and ASWBF colonies consistently displayed an order of magnitude difference in aligned/tilted domain ratio, the method was considered reliable.

In addition to a shift in wavelength and angular distribution, the spectra of fucoidan samples in figure 2*a* also show a significant decrease in intensity of the reflected light. This can be attributed to a loss in the ordering of the cells. This observation is confirmed by the analysis of individual domains performed via dark-field optical microscopy and cryogenic scanning electron microscopy. We, in fact, observe that the fucoidan-grown colonies have a smaller average domain size and less local ordering, decreasing the reflected light intensity (electronic supplementary material, figure S9 and S10). The weak local ordering could in part be caused by the greater variation in cell diameter for fucoidan-grown samples (electronic supplementary material, figure S8), as cells of different sizes will not be able to pack efficiently.

The combination of the optical and anatomical studies of the colonies therefore suggests that the cellular organization is strongly affected by the presence of fucoidan, which not only increases the diameter of the cells, thereby changing the colour of the colony but also perturbs the otherwise highly ordered organization on the cells. Moreover, cell motility is also affected: on ASWBF plates, IR1 displays a ring-like expansion, reminiscent of the biphasic expansion observed in the closely related strain *Flavobacterium* *johnsoniae*, when grown on the polysaccharide pectin [32]. Such behaviour stands in sharp contrast to the homogeneous spreading behaviour observed on ASWB and ASWBC plates (electronic supplementary material, figure S7b). Interestingly, these effects are not observed for IR1 colonies grown in the presence of *κ*-carrageenan.

### 2.3. Fucoidan effects link to a specific metabolic pathway

**Figure 3.**
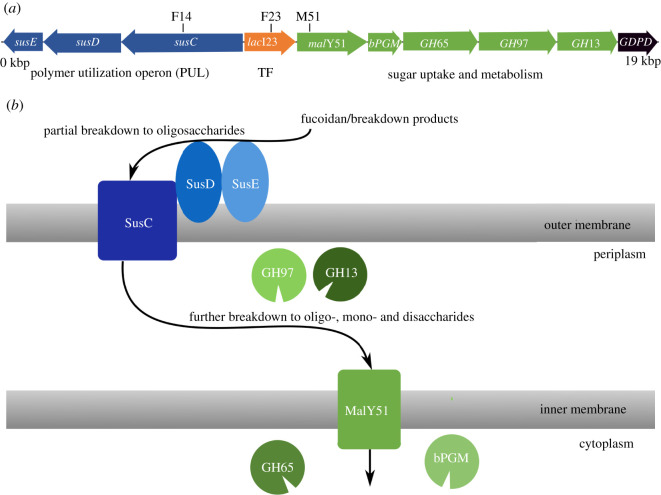
(*a*) Map of the region of the IR1 chromosome where transposon insertions in IR1 affecting polysaccharide degradation and structural colour were mapped by DNA sequencing. Genes encoding known and predicted functionalities are colour-coded (from left to right): cluster of genes of a polymer utilization operon (PUL) *sus*E, *sus*D and *sus*C, are represented with blue arrows. Transcriptional factor (TF) *lac*I23, in orange. Enzymes that participate in sugar and starch metabolism in green, maltose transporter protein, *mal*Y51; beta-phosphoglucomutase enzyme, *bPGM*; glycoside hydrolases family 65, 97 (alpha-glucosidase) and 13 (alpha-amylase) proteins (*GH*65, *GH*97, *GH*13). Glycerophosphoryl diester phosphodiesterase enzyme, *GDPD,* in black. Black lines above the genes indicate the position of transposon insertions. (*b*) Schematic overview of the biochemical pathway encoded by the relevant genes indicated in (*a*).

To better understand how fucoidan expresses its influence on the organization of IR1 cells, we investigated the biochemical pathways involved. We studied a series of mutants, each of which have transposon insertions in a single point in the chromosome, disrupting the function of one or more genes. These mutants were selected by screening a transposon library [25] for colonies that no longer showed fucoidan-mediated colour changes. Multiple, independently isolated mutants were found to map within the closely clustered set of genes described below, with identical phenotypes (figure 3*a*; electronic supplementary material, figure S11 and table S1). Mutants F14, F23 and M51 were chosen to be analysed further.

Mutant F14 has a transposon insertion into the upstream gene of a polysaccharide utilization locus (PUL) operon, within the SusC protein (*sus*C, figure 3*a*). PUL operons are commonly involved in the binding, uptake and early stage metabolism of polysaccharides [33–35]. The genes downstream of *sus*C (*sus*D/E) bind polysaccharides and may perform a partial breakdown of the polysaccharides into oligosaccharides (figure 3*b*). SusC then is expected to transport these products across the outer membrane into the periplasm, where they can be metabolized further.

Mutant F23 has a transposon insertion in a gene encoding a transcription factor (TF) that is involved in sugar metabolism (*lac*I23, figure 3). A predicted gene encoding a maltose transporter protein (*mal*Y51) is located downstream of the TF, which was identified in the mutant screening as mutant M51, and it is followed by several enzymes putatively involved in the metabolism of maltose and related sugars (*bPGM*, *GH*65, *GH*97 and *GH*13). These proteins are relevant as maltose is a breakdown product of fucoidan, and can be expected to act on a wider range of sugars, some of which may also be degradation products of fucoidan [30].

**Figure 4.**
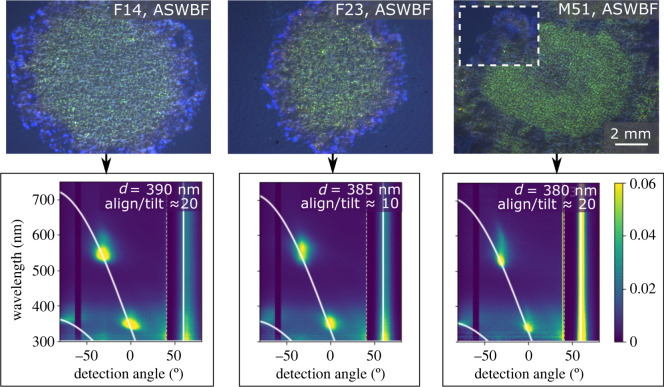
Optical appearance of metabolic mutants, grown in the presence of fucoidan (ASWBF, day 1 of growth). The images in the top row show the centre of each colony, at the location where the initial cell suspension was deposited. The inset for M51 shows the edge of each colony at the same scale as the main images. Images are taken with a stereo microscope. The bottom row shows the angular resolved scattered light spectra corresponding to the images above, taken at an incident light angle of −60°.

We find that none of the mutants display the striking fucoidan-specific optical effects observed with the WT (figure 4; electronic supplementary material, figures S12, S13 and S14). The lattice constant for mutant colonies on ASWBF plates and the ratio of aligned to tilted domains is consistently at a value comparable with the WT on ASWB plates, which is in accordance with the green colour they display. Moreover, apart from a small spectral smear that is also observed for the WT strain, which can be explained by slight variations in the lattice constant, we found none of the other types of disorder that is normally observed in presence of fucoidan (electronic supplementary material, figure S15 and S16), leading to an unvaried bright green iridescent appearance, as can be seen in figure 4.

Therefore, we conclude that the biochemical processes involved in the fucoidan-related response include the binding of polysaccharides to the outside of the cell, their processing and uptake into the periplasm and transport of sugars such as maltose into the cytoplasm, as shown in figure 3*b*. While it is quite possible that transposon insertions we have studied may have polar effects, this does not substantially affect these conclusions. Such an observation, in turn, implies that the uptake and metabolism of fucoidan is required to trigger the observed optical response rather than any independent sensory process, such as a receptor on the cell surface that triggers a behavioural response.

It is particularly striking that in this metabolic pathway a single region of the chromosome has such a major influence on how fucoidan modulates photonic structures in IR1. Such an observation is even more remarkable, considering that IR1 has at least 11 PUL operons and yet mutants were only found within the one described. Fucoidan, therefore, does not only affect the regulation of the cellular organization in IR1, but it does so using a highly specific metabolic pathway, implying that the effects of fucoidan are not simply the result of a general polysaccharide metabolism, but that the regulatory pathways involved are connected to this specific compound. This is further confirmed by our observations on *κ*-carrageenan: IR1 barely responds to the addition of *κ*-carrageenan in terms of cellular organization, and where it does have an effect it does not resemble the changes seen for fucoidan.

While it remains unclear what the benefits of the fucoidan-triggered adaptation of the cellular organization could be, the specificity of the biochemical pathway involved suggests that the response is not an accidental by-product of a general polysaccharide metabolism, and that there is a purpose to the uptake of fucoidan. Moreover, it supports the cohabitation of IR1 with brown algae or with products derived from brown algae. This could potentially indicate a more complex functionality of the response, such as a symbiotic mechanism between IR1 and certain macroalgae.

## 3. Conclusion

We have found that cells of the structurally coloured bacterial strain IR1 change their organization, and therefore the optical appearance of the colony, in response to the polysaccharide fucoidan. We have characterized this response in detail, showing that the diameter of the cells increases, changing the colour of the colony, and that the crystalline organization of the cells becomes more disordered. By analysis of mutant strains we have revealed a metabolic pathway that is involved in this effect. As such, we have demonstrated not only that the uptake and metabolism of fucoidan are required for the optical effect to occur, but also that this pathway is highly specific, using only one out of multiple PUL operons available. This sensitivity to fucoidan implies that IR1 shares its natural habitat with brown algae, and that the bacteria respond to the presence of the algae by changing their cell organization. This latest observation could have significant implications for the function of the structural organization in the *Flavobacteriia*, most of which commonly metabolize algal polysaccharides.

## 4. Material and methods

### 4.1. Bacteria culture conditions

Strain IR1 was isolated on ASWB agar plates (table 1) during a screening of estuarine sediment samples from the brackish water from the Neckarhaven region of Rotterdam harbour [25]. IR1 is a yellow-pigmented Gram-negative bacterium culturable under aerobic conditions from 2 to 30°C. Agar plates were prepared as follows: agar medium containing nutrients as outlined in table 1 was sterilized using an autoclave (Prestige Medical classic). Plates were poured directly after the autoclave cycle has finished into plastic Petri dishes (90 mm Triple vent, Appleton), and then left to air-dry for 30 min in a class II safety cabinet. After drying, plates were closed and subsequently sealed off with parafilm. They were stored at 4°C until further use.

Inoculation of the bacteria was performed by suspending cells from a healthy colony grown on ASWB plates in a saline solution (1% (w/v) KCl in Milli Q water), until the suspension reached an absorbance of 0.127 at 600 nm (measured using a Jenway 6300 spectrophotometer). Five millilitres of this suspension was then deposited in the centre of each plate, which were subsequently left to air-dry for 15 min in a class II safety cabinet before closing the Petri dishes and sealing them off with parafilm. All bacteria were subsequently grown in the dark at 25°.

### 4.2. Identification of transposon insertion sites

A transposon library was created, as previously described [25,34], and screened for colonies that remained an iridescent green on ASWBF agar plates, unlike the red/purple coloration of the WT. Mapping of the transposon insertion for M51 and F14 mutants was described by Johansen *et al.* [25]. Whole-genome sequencing was performed for mutants F5, F13, F21 and F23. DNA libraries were generated, and the paired ends were sequenced 2 × 150 bp using Illumina Hiseq (GATC Biotech, Germany). Reads from Illumina Hiseq were analysed and transposon flanking insertion regions were identified using Geneious R v. 9.1 software (Biomatters Ltd, Auckland, New Zealand [36] (http://www.geneious.com).

### 4.3. Angular resolved optical spectroscopy

Angular resolved spectra were taken using a custom-built goniometer set-up [29]. Samples were prepared by cutting out a piece of roughly 1 cm^2^ out of the agar. This piece was subsequently attached to a microscope slide with double-sided tape. The slide containing the bacteria on agar was then mounted on a rotating stage, and illuminated using light in the UV–VIS region (Ocean Optics HPX-2000 xenon lamp) that was collimated through an optical fibre that was attached to a reflective collimator (Thorlabs RC08SMA-F01). This resulted in an illuminating light beam with a spot size of 5 mm diameter. The angle of incidence of this light could be varied by rotating the sample. Light that was reflected and scattered from the sample was collected using the same fibre–collimator combination as used for the incident beam. The fibre was subsequently connected to a spectrometer (AvaSpec-HS2048, Avantes). The detection fibre and collimator were mounted on a rotating arm so that the angle of detection could be varied. At the detection angle, which equals the negative of the incident angle, no signal was collected due to a limitation in the set-up. All the spectra reported here were normalized against a white diffuser (labsphere SRS-99-010) at 0° incident and 5° collection angle.

### 4.4. Analysis of specular reflection spectra

The angular resolved specular reflection signal, as obtained by goniometry measurements, can be used to extract the average refractive index of the sample (*n*_avg_), as described in detail in [11]. This can be done by fitting *n*_avg_ using the following equation:

4.1 $$\lambda_{s}=\lambda_{p}\cos(\theta_{2})=\lambda_{p}\cos\left(\arcsin\frac{\sin\theta_{\mathrm{in}}}{n_{\mathrm{avg}}}\right).$$

Here, *λ*_*p*_ is the wavelength of the peak reflection at normal incidence, which is shifted in angular space to *λ*_*s*_. *θ*_2_ is the angle at which light waves are travelling through the sample (as given by Snell’s Law) and *θ*_in_ is the angle of incidence of the light.

### 4.5. Analysis of scattering response

Light reflected off the bacterial colony shows up as diffraction spots in the angular resolved scattered light spectra. As previously described [11], we can retrieve the lattice constant of the crystal structure by fitting these spots to the grating equation

4.2 $$\theta_{m}=\arcsin\left(\frac{m\lambda }{d}-\sin\theta_{i}\right),$$

where *m* is an integer indicating the diffraction order, *λ* the wavelength of the reflected light, *θ*_*m*_ and *θ*_*i*_ are the angles of the reflected and incident light, respectively, and *d* the period of the structure.

### 4.6. Optical microscopy

A stereo microscope (Zeiss stemi 305) was used to show the overall optical appearance of the bacteria (figures 1 and 3; electronic supplementary material, figure S12). Other optical microscope images (electronic supplementary material, figures S9, S10, S15 and S16) were taken with a Zeiss Axio Scope A.1.

### 4.7. Cryogenic scanning electron microscopy

Cryogenic scanning electron microscopy (Cryo-SEM) images were obtained using an FEI Verios 460 scanning electron microscope, equipped with an Everhart–Thornley detector and with a Quorum cryo-transfer system P3010T at the Cambridge Advanced Imaging Centre. A piece of agar with the bacterial colony on it was cut out from the plate and attached to a specimen holder with colloidal graphite suspension. The specimen holder with the sample was then plunge frozen in liquid ethane and transferred to a preparation chamber that was cooled down to −140°C. The sample was then sublimed at −90°C for 2 min, sputter-coated with platinum, and imaged at 2.00 kV acceleration voltage with 25 pA probe current.

### 4.8. Cryogenic transmission electron microscopy

Cryo-TEM images were obtained using a Thermo Scientific (FEI Company) Talos F200X G2 microscope operating at 200 kV. Images were recorded at low dose using a Ceta 4k × 4k CMOS camera and Velox software. Specimens were vitrified by plunge freezing an aqueous suspension of bacteria (with salinity of 0.01% KCl) onto copper grids (300 mesh) with lacey carbon film. The sample was transferred onto the TEM grid, blotted for 3 s at blot force −10 using dedicated filter paper, and immediately frozen by plunging into liquid ethane using a fully automated and environmental controlled blotting device, Vitrobot Mark IV. The vitrobot chamber was set to 4°C and 95% humidity. After vitrification the specimens were kept under liquid nitrogen until they were inserted into a Gatan Elsa cryo holder and imaged in the TEM at −178°C.

### 4.9. Domain size image analysis

Optical microscopy images, taken with a Zeiss Axio Scope A.1, were processed using the openCV library implemented via a custom Python script. Non-local means denoizing was used to remove speckle noise from the camera, before converting the images to greyscale. The two-dimensional power spectral density was calculated using the fast Fourier transform and averaged over all azimuthal angles. The analysis was done on 15 images per nutrient condition (day 1 of growth, NA 0.3), and images were taken from different central areas on three plates per nutrient condition.

## Data Availability

All data needed to evaluate the conclusions in the paper are present in the paper and/or the electronic supplementary material are available at the University of Cambridge data repository https://doi.org/10.17863/CAM.72481 [37].

## Endnote

1 The measured angle of the primary diffraction peak is highly sensitive to the alignment of the sample in the angular-resolved spectroscopy set-up.
